# Real-time 3D single-molecule localization microscopy analysis using lookup tables

**DOI:** 10.1364/BOE.424016

**Published:** 2021-07-16

**Authors:** Fabian Hauser, Jaroslaw Jacak

**Affiliations:** 1 University of Applied Sciences, Upper Austria School of Medical Engineering and Applied Social Sciences, Garnisonstraße 21, 4020 Linz, Austria; 2 Austrian Cluster for Tissue Regeneration, Vienna 1200, Austria

## Abstract

Herein, we present a new algorithm for real-time analysis of 3D single molecule localization microscopy images with a small impact on fitting accuracy using lookup-tables with discrete xyz-positions. The algorithm realizes real-time visualization during acquisition. We demonstrate its performance on simulated and measured data. Additionally, combining real-time fitting with a feedback loop controlling the activation laser pulse keeps the number of emitters per image frame constant.

## 1. Introduction

In recent years, Single Molecule Localization Microscopy (SMLM) has gained popularity due to commercially available whole-packaged systems and a wide range of freely available analysis software. In particular, direct Stochastic Optical Reconstruction Microscopy (dSTORM [1]) using photoswitching of fluorophores with a single laser wavelength and allowing modulation of the Point Spread Function (PSF), enables 3D applications by extracting the axial position from the signal’s shape [2–5].

Typically, tens of thousands of images are required to reconstruct a single super-resolved image. Only a random subpopulation of sparsely distributed fluorophores is observable in each frame. Sparsity is needed to distinguish among emitters to enable precise localization. During the analysis, each frame is post-processed to obtain subpixel position of the fluorescence signal. Nevertheless, there are disadvantages if the images are analyzed after acquisition: (i) analysis of image stacks is time-consuming and can take up to tens of minutes depending on the amount of frames and emitters per frame, (ii) several parameters such as illumination time, laser power, pH-value, density of active fluorophores, and photo-switching cycle times influence blinking quality [6–8]. The impact of these parameters can only be evaluated after experimental data is analyzed. In contrast, using real-time SMLM analysis, some of these parameters can be adjusted during the acquisition phase to obtain an optimal emitter density per frame, e.g. by using an additional ultraviolet (UV) laser pulse which reactivates fluorophores from their dark-state. Many software packages already offer real-time analysis of SMLM data through the use of either multiple central processing unit (CPU) cores/threads [9], graphics card acceleration (using graphics processing unit (GPU)) [10–14] or experimental PSFs [15] in combination with a CPU and GPU. However, high-end CPUs/GPUs are expensive and/or are often not available on the computer used for the measurement.

In this paper, we present a real-time SMLM algorithm, which only requires a single CPU core to perform fitting of up to 600 astigmatic single emitters within 9.8 ± 0.5 milliseconds (ms) per frame – achieved by using a lookup table generated prior to the experiment. We verified our algorithm with simulated data, tested it on tubulin-like simulations from the SMLM challenge 2016 [16] and compared it to published results of the challenge. Our algorithm scored at mid-field with an efficiency of 61%. We further tested our algorithm on simulations of 3D Siemens star-shaped test patterns [10]. Likewise, our algorithm localizes emitters at different axial positions; only extremely fine structures with axial positions below z < −400 nm were not detectable. Additionally, we tested the algorithm on data obtained from fixed biological samples and imaged the actin distribution in human endothelial cells and human platelets. Finally, we compared the spatial and temporal performance of our algorithm with our continuous least-squares fitting algorithm (3D STORM Tools [17]) as well as the optical resolution of the rendered localization microscopy images using Fourier Ring Correlation (FRC). Our real-time lookup table algorithm only requires on average 2.5 ± 0.6 ms to analyze an image frame while at the same time using only a single CPU core. This short analysis time allows for rendering of the SMLM image in real-time and for control of the number of active fluorophores per frame using an activation laser pulse.

## 2. Materials and methods

### 2.1. Real-time fitting of diffraction limited signal of fluorescent emitters

Our algorithm is based on a lookup table generated prior to the experiment. First, the lookup table is populated with template images of a PSF model at discrete lateral/axial positions and their derivatives ∂PSF(x,y,z)/∂x, ∂PSF(x,y,z)/∂y, ∂PSF(x,y,z)/∂z needed for fitting (see Visualization 1 for a movie of a populated lookup table). Hereby, any PSF model can be chosen as long as the PSF and first derivatives exist. For example, we use a 2D elliptical Gaussian function (Eqs. 1 & S1-S2 in Supplement 1). Here, φ is the clockwise rotation of the 2D Gaussian model, *σ_x_(z)* and *σ_y_(z)* are values of the fitted calibration using cubic B-spline [18]. The parameters *x*- and *y-position* are varied over a range of *r_xy_* = 4 pixels around the center of the template image using Δ*xy* = 0.1 pixels steps. Next, the elliptical Gaussian shapes (retrieved from calibration) representing axial positions of emitters are varied over a range of *r_z_* = 1000 nm in Δ*z* = 25 nm steps. At each discrete position of the lookup table, the PSF model and derivatives are calculated at each i^th^ and j^th^ pixel inside a 9 × 9 pixels template image. A lookup table with the parameters *window size* = 9 pixels, Δ*xy* = 0.1 pixels, *r_xy_* = 4 pixels, Δ*z* = 25 nm, *r_z_* = 1000 nm uses 263 MB of RAM with double precision values and 106 641 unique template images (each 4-dimensional pixel of the template image contains the PSF and their first derivatives of xyz).

(1) $$\begin{array}{ll} PSF_{i,j}(x,y,z,\varphi ) & =\text{exp}(-\frac{{\left[\left(i-x)\text{cos}(\varphi )+(j-y)\text{sin}(\varphi \right)\right]}^{2}}{2\sigma_{x}{\left(z\right)}^{2}}\\ & -\frac{{\left[-(i-x)\text{sin}(\varphi )+(j-y)\text{cos}(\varphi )\right]}^{2}}{2\sigma_{y}{\left(z\right)}^{2}}) \end{array}$$

In order to find localization candidates, a modified Non-Maximum Suppression (NMS) algorithm [19] is used. The NMS algorithm is modified in a way that a mean of nine pixels which surround an identified maxima was used to suppress non-maxima, and not as usually a single pixel [19]. Next, the local background is calculated by averaging the pixel intensities along a square boundary line of a predefined window (e.g. 10 × 10 pixels). By comparing these values with a predefined intensity threshold, the chance of finding a wrong maximum is further decreased. Each fit is performed in a window surrounding the identified candidate, the window size matched the template images from the lookup table. Our recent NMS implementation is not suited for overlapping emitters. Therefore, our experiments are designed to have the smallest possible amount of overlapping emitters.

For fitting, we use an iterative Gauss-Newton algorithm to minimize the parameters of our discrete model: the parameters *θ_bg_* (mean background signal), *θ_p_* (maximum intensity) are unconstrained and the parameters *θ_x_,θ_y_,θ_z_* are constrained by the discrete positions used by the lookup table. The least-square (LS) minimization method is chosen instead of maximum-likelihood estimator (MLE) [20], since only the first derivatives are required. In order to find the best fit for a single emitter, we first define a window around the candidate’s position matching the window size of the lookup table’s template. Next, we calculate the Jacobian matrix elements *J_i,j_* (Eq. (2)) at every position within the window based on the template at the proposed position from the initial vector $\vec{\theta }=(\theta_{bg},\theta_{p},\theta_{x},\theta_{y},\theta_{z})$. The initial values of *θ_bg_* and *θ_p_* are derived from the candidate search of the modified NMS algorithm and *θ_x_,θ_y_,θ_z_* are initialized to fixed values (*θ_x_,θ_y_* to the center of the fitting window and θ_z_ to the focal plane with maximum focus found during calibration).

(2) $$J_{i,j}(\vec{\theta },k)=\left(\begin{array}{c} 1\\ PFA_{i,j}^{\left(LUT\right)}(k)\\ \theta_{p}\frac{\partial PFA_{i,j}^{\left(LUT\right)}(k)}{\partial \theta_{x}}\\ \theta_{p}\frac{\partial PFA_{i,j}^{\left(LUT\right)}(k)}{\partial \theta_{y}}\\ \theta_{p}\frac{\partial PFA_{i,j}^{\left(LUT\right)}(k)}{\partial \theta_{z}} \end{array}\right)$$

We calculate the index *k(θ_x_,θ_y_,θ_z_)* of the template image in the lookup table using positions *θ_x_,θ_y_,θ_z_*. Based on the obtained index, we subtract the scaled and offset model from the observed data (*I_i,j_)* within the candidate window to get the residual vector *r_i,j_*:

(3) $$r_{i,j}(\vec{\theta },k)=I_{i,j}-(\theta_{bg}+\theta_{p}\cdot PFA_{i,j}^{\left(LUT\right)}(k))$$

We use the LAPACK (Linear Algebra Package) library [21] to speed up calculation for finding the next parameters (Eq. (4)), especially **syrk** (symmetrical rank-k matrix multiplication) for $J^{T}J$ and **trsv** (solves for a triangular system of equations) to solve the equation system for ${\left(J^{T}J\right)}^{-1}Jr$.

(4) $${\vec{\theta }}^{\left(s+1\right)}={\vec{\theta }}^{\left(s\right)}+{\left(J_{i,j}^{T}J_{i,j}\right)}^{-1}J_{i,j}r_{i,j}$$

Since $J^{T}J$ is a symmetrical matrix, it is faster to solve the equation system directly from the triangular matrix rather than to perform a Cholesky decomposition [22] with consequently solving the equation system. Next, the parameters ${\vec{\theta }}^{\left(s+1\right)}$ are tested for convergence. For further iterations, the parameters *θ_x_,θ_y_,θ_z_* are rounded to the next valid step based on the lookup table parameters Δ*xy* and Δ*z*, hence the index *k(θ_x_,θ_y_,θ_z_)* is updated. These calculations (Eq. (4)) are repeated until either the maximum number of iterations is reached,*θ_x_,θ_y_* are outside of *r_xy_*,*θ_z_* is outside of *r_z_*, or convergence is reached.

### 2.2. Human platelet concentrates

All human blood samples were collected during routine plateletpheresis in accordance with the strict policies of the Red Cross Transfusion Service, Linz, Austria. All blood donors signed informed consents stating that residual blood material could be used for research and development purposes. All experimental protocols were approved by and carried out in collaboration with the Red Cross Blood Transfusion Service. Single donor platelet concentrates were provided by the Red Cross Blood Transfusion Service. Platelet concentrates were prepared by apheresis with an automated cell separator (Trima Accel Automated Blood Collection System, TerumoBCT, Lakewood, CA, USA) during routine plateletpheresis: platelets were separated from whole blood by centrifugation and diluted in 35% plasma, 65% platelet additive solution SSP+ (Macopharma, Mouvaux, France), and ACD-A anticoagulant (Haemonetics anticoagulant citrate dextrose solution, Haemonetics, Braintree, MA, USA) during the transfer into Trima Accel storage bags. Two milliliters of the platelet concentrate (typically containing 1 × 10^6^ platelets/µL) were transferred to a new storage bag and immediately transported to the laboratory. Transportation within a polystyrene box minimized temperature variations. Platelets were used for experiments within 24 h after preparation and stored under constant agitation in a climatic chamber that was set to 22 °C.

### 2.3. Platelet staining

Platelets were diluted to a final concentration of 2 × 10^4^ cells/mL in cell culture medium (DMEM, Sigma-Aldrich, Vienna, Austria) and were allowed to settle on a glass slide for 15 min. Non-adherent cells were washed away with Phosphate-Buffered Saline (PBS). Actin cytoskeleton was visualized using Alexa Fluor 647 phalloidin (Cell Signaling Technology, Leiden, The Netherlands) in a Cytoskeleton Buffer with Sucrose (CBS) containing 10 mM MES pH 6.1, 138 mM KCl, 3 mM MgCl_2_, 2 mM EGTA, and 0.32 M sucrose according to a protocol from Louise Cramer [23] (MRC Laboratory for Molecular Cell Biology, UCL, London, UK). Briefly, platelets were fixed using 4% paraformaldehyde in CBS for 20 min at room temperature, then permeabilized using 0.5% Triton X-100 with CBS, blocked in 10% chicken-egg-white-albumin (Sigma-Aldrich, Vienna, Austria) and stained for 20 min with 66 nM Alexa Fluor 647 conjugated to phalloidin.

### 2.4. Human endothelial cells CD34^+^-EC

Primary human Endothelial cells (phECs) were differentiated from CD34^+^ cells isolated from human cord blood as previously described [24] and were provided in frozen aliquots of 1 million cells at passage 5 by Prof. Gosselet, Université d´Artois, France. After thawing, cells were seeded onto gelatine (0.2% in PBS) coated 10 cm-dishes in ECM-5 medium (ECM from Sciencell with 5 mL of Endothelial cell growth supplement (ECGS), 2.5 mL of Gentamycin 10 mg/mL (BiochromAG, Ref. A-2712) and 25 mL of pre-selected, heat-inactivated FBS; and cultivated at 37 °C, 5% CO_2_. When cells reached confluency, they were washed three times with PBS, detached with Trypsin/EDTA solution, counted and re-seeded at a concentration of ∼20 000 cells/cm^2^. Expression of EC marker CD31 was confirmed by flow cytometry and immunofluorescence.

### 2.5. Endothelial cell staining

Cells were split and seeded at approximately 20 000 cells/cm^2^ into Nunc Lab-Tek II Chambered Coverglasses (Thermo Fisher Scientific Inc, MA, USA). Next, cells were washed with pre-warmed HBSS (containing Mg^2+^ and Ca^2+^) at 37 °C. The actin cytoskeleton was visualized using Alexa Fluor 647 phalloidin (Cell Signaling Technology, Leiden, Netherlands). Staining of the cells was conducted in CBS as described in the platelet staining section.

### 2.6. Fluorescence microscope

Images were acquired using a modified Olympus IX81 inverted epifluorescence microscope with an oil-immersion objective (PlanApo N 60x/1.42 NA, Olympus, Vienna, Austria) as well as an additional tube-lens with a magnification of 1.6x. The sample was positioned on a XYZ piezo stage (200 µm x 200 µm x 200 µm range, P-562.3CD, Physical Instruments) on top of a motorized stage with a range of approximately 1 cm × 1 cm (HybridStage, JPK Instruments, Berlin, Germany). Fluorescent signals were detected using an Andor iXonEM+ 897 (back-illuminated) electron multiplying charge coupled device (EMCCD) camera (16 µm pixel size, Andor Technology, Belfast, Northern Ireland). This results in an image pixel-size of 166.7 nm/pixel and a total magnification of 96x. Fluorescently labeled samples were excited using a 640 nm solid-state laser (diode-pumped, iBeam Smart, Toptica Photonics Gräfelfing, Germany); and under certain conditions fluorophores were additionally recovered from dark-state with a 405 nm diode laser pulse (iPulse, Toptica Photonics Gräfelfing, Germany). An additional cylindrical lens (f = 500 mm, Thorlabs, Newton, NJ) was introduced in the pathway between camera and the microscope’s side port for 3D single-molecule localization microscopy. Fluorescence emission was additionally filtered with a 700/50 nm emission bandpass filter (AHF, Tübingen, Germany).

### 2.7. dSTORM

3D dSTORM experiments were performed in a medium containing 50 mM β-mercapto-ethylamine (MEA), 30% glycerine and PBS, a region of interest (ROI) of 256 × 256 pixels and 10 000 frames were acquired. Fluorophores were illuminated for 20 ms at each frame and an optional 20 ms UV illumination pulse during the readout time of the CCD camera. Prior to each experiment, a calibration for the emitter’s axial position localization (compensating the axial PSF distortion) was performed using TetraSpeck (0.1 µm, Thermo Fisher Scientific) microspheres. Experiments were analyzed using the presented real-time lookup table algorithms or by fitting a constrained (σ_x_ & σ_y_) continuous elliptical Gaussian function as previously reported [17]. Typically, position fits were continuous, whereas the lookup table algorithm utilized quantified steps (e.g. 0.1 pixels depending on the parameters for the lookup table generation).

### 2.8. Computation system architecture

Computations for simulated datasets were performed on a notebook (CPU: Intel Core i7-8650U with 4 cores at 1.9GHz, 32 GB RAM, Window 10 Education operating system 64 bit) and cell measurements were performed at a workstation (CPU: Intel Xeon CPU E3-1271 with 4 cores at 3.6 GHz, 16 GB RAM, Window 7 Professional operating system 64 bit) equipped with the hardware to control a 405 nm laser and the EMCCD camera.

## 3. Results

Here, we present a 3D real-time SMLM algorithm not depending on GPU acceleration nor multithreading. We show that lookup tables of pre-calculated 2D elliptical Gaussian signals which approximate the PSF for various lateral positions and shapes (width and height of 2D elliptical Gaussian functions) can be used to accelerate the fitting of fluorescent emitters (approximately 10 times faster compared to 3D STORM Tools [17]).

### 3.1. Analysis of simulated data

**Fig. 1.**
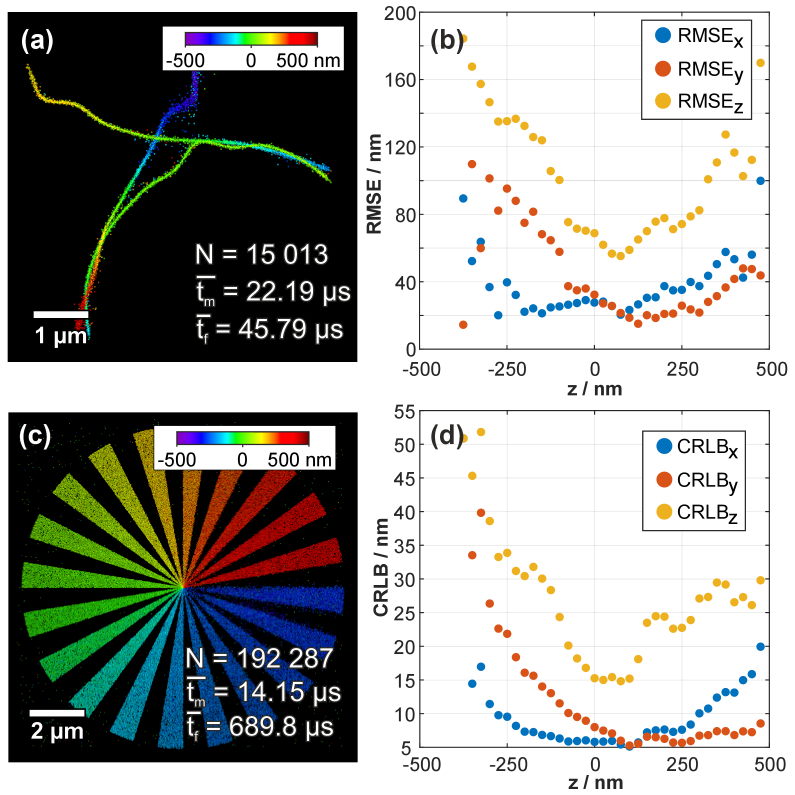
**Lookup table algorithm application on simulated SMLM datasets.** The lookup table was populated with templates of 2D elliptical Gaussian functions which approximate the PSF with a window size of 11 pixels and 9 pixels for (**a**) and (**c**) respectively with different lateral positions at 0.1 pixel steps (equals 10 nm steps) and different shapes depending on the axial position over a range of 1000 nm in 25 nm steps. In total, 106 641 different templates were generated. (a) Three-dimensional simulation of microtubules from the single-molecule localization microscopy symposium challenge. The training dataset *MT0.N1.LD* was used to test the lookup table algorithm and determine the 3D position of emitters, where on average a single emitter intensity fit need *t_m_* = 22.19 µs, a frame including fitting needed on average *t_f_* = 45.79 µs and a total of *N* = 15 013 emitters was fitted. A comparison of fitted positions with the ground truth dataset, using the comparison tool provided by the challenge, resulted in a Jaccard index of 65%, lateral and axial RMSE of 45 nm and 76 nm respectively, an intersection of 14 899 molecules and an efficiency of 40%. (b) shows the comparison of lateral and axial RMSE to the axial position retrieved from the comparison tool provided by the SMLMS challenge 2016. Our fitted positions are compared to the ground truth of the challenge simulated dataset in (**a**). (c) Simulation of a 3D test pattern made up of 20 circle sectors of increasing axial position and 20 blank circle sectors in between. The axial spoke steps reach from -450 nm to 450 nm in 45 nm steps size. Emitter axial positions are normally distributed for each axial steps with a sigma = 25 nm. The analysis using our lookup table algorithm resulted in an average time to fit a single emitter signal of *t_m_* = 14.15 µs, a frame including fitting needed on average *t_f_* = 680.8 µs and a total of N = 192 287 emitters was fitted. (**d**) Mean values of the Cramér–Rao lower bound (CRLB) values determined from the discrete z-position (25 nm steps) of the fitted SMLMS 2016 challenge dataset positions.

In order to evaluate the performance of our lookup-based algorithm, simulations were generated and analyzed to determine axial and lateral precisions. As a reliable source for SMLM simulations, the 3D microtubule-like datasets [16] from the single-molecule localization microscopy symposium (SMLMS) challenge 2016 organized by École Polytechnique Fédérale de Lausanne (EPFL) were chosen. One modality of the challenge was 3D astigmatism (http://bigwww.epfl.ch/smlm/challenge2016/datasets), which we used for evaluation (see Fig. 1(a)). The analysis of the training dataset *MT0.Nx.LD* by our lookup table algorithm required 12 seconds to complete on a single CPU core. The lookup table was populated with templates of 2D elliptical Gaussian functions that approximate the PSF. A window size of 11 pixels was chosen and the lateral positions were varied over a range of *r_xy_* = 4 pixels around the center in both directions at Δ*xy* = 0.1 pixel steps (equals 10 nm steps for an image pixel size of 100 nm). In addition to the lateral position variation, different shapes of the PSF corresponding to axial positions over a range of *r_z_* = 1000 nm in Δ*z* = 25 nm steps were generated. In total 106 641 unique template images were generated. We compared the temporal performance to the continuous least-squares fitting algorithm of our 3D STORM Tools software [17], which needed 20 seconds to fit 20 000 frames on four (+4 virtual) CPU cores. Next, we evaluated the spatial performance of the lookup table algorithm by comparing the ground truth and analyzed dataset. We used the software provided by the SMLM challenge (*CompareLocalization*) to extract the evaluation values as previously described [16]. Furthermore, we compared the lateral and axial root mean squared localization error (RMSE) over the axial range (see Fig. 1(b)). We scored a Jaccard index [25] of 65% for the high Signal-to-Noise-Ratio (SNR) and 50% for the low SNR, lateral RMSE of 45 nm for the high SNR and 56 nm for the low SNR, axial RMSE of 76 nm for the high SNR and 92 nm for the low SNR, an intersection of 14 899 molecules for the high SNR and 11 451 for the low SNR with an overall efficiency of 61% (40% for the high SNR and 21% for low the SNR).

Additionally, we calculated the Cramér-Rao lower bound (CRLB) for each fitted single emitter signal (see Fig. 1(d)). The CRLB is the “fundamental theoretical limit of localization precisions obtained by unbiased estimator” as previously stated [26]. This lower limit for localization precision can be reached by using maximum-likelihood estimator (MLE) due to the Poisson noise distribution of photon emission. However, we used a least-square estimation for emitter fitting and only calculate the CRLB as measure for lowest possible positional accuracy. In Fig. S1 we present the fitting accuracy over multiple frames and CRLB values for imaged fluorescent beads in different axial positions.

Next, we tested the influence of lower lateral and axial step sizes for the lookup table. Here, we chose a lateral step size of Δ*xy* = 0.05 pixel (corresponding to 5 nm steps) and an axial step size of Δ*z* = 10 nm and a window size of 9 pixel. The lateral RMSE decreased by 1.26% (-0.64 nm absolute), the axial RSME by 2.89% (-2.53 nm absolute) and the overall efficiency increased by 2% to 61%. These small incremental changes were not sufficient to justify the greatly increased RAM usage which went from 264 MB to 2.49 GB for the lookup table.

Furthermore, we tested a PSF model, which takes into account pixelation of the EMCCD camera. This model is based on integration of a 2D elliptical Gaussian function [20,27]. The only changes to the model were introduction of our cubic B-spline values for *σ_x_(z)* and *σ_y_(z)* instead of the polynomials. Furthermore, we multiplied the values of the model with *2πσ_x_(z)σ_y_(z)* to convert the integrated intensities to maximum peak intensities (equations S3, S4). We analyzed the SMLMS challenge 2016 dataset (low and high SNR) and compared it to our default 2D elliptical Gaussian model (window size 9 pixels, Δ*xy* = 0.1 pixels, *r_xy_* = 4 pixels, Δ*z* = 25 nm, *r_z_* = 1000 nm). The lateral RMSE decreased by -1.26% (-0.14 nm absolute) whereas the axial RSME increased by +1.02% (+0.89 nm absolute) and the overall efficiency increased only by 1% to 60%. Furthermore, our default 2D elliptical Gaussian model needed 14.3 ms ± 1.1 ms (N = 1000 repeats) to generate the lookup table model, the integrated Gaussian model needed 44.2 ms ± 3.0 ms (N = 1000 repeats).

Some defocused emitters near the axial boundaries (see Fig. 1(b)) of the challenge dataset could not be detected. We further investigated these boundary cases with an additional simulated dataset. We simulated a 3D Siemens star-shaped test pattern [10] with discrete axial steps of 45 nm starting from -450 nm to 450 nm (see Fig. 1(c)). The axial position of each emitter was normally distributed around each step with a standard deviation of 25 nm. The pattern was made up of 40 spokes consisting of 20 circle sectors of increasing axial position and 20 blank sectors in between. Simulated emitters were rendered using a 2D elliptical Gaussian function to approximate real PSFs and an ellipticity depending on the axial position (gained from the calibration experiment). Furthermore, noise was added to each simulated frame including readout noise, electron-multiplying noise, and clock-induced charges (baseline: 100 counts, mean peak intensity: 2000 counts, background: 0 counts, EM gain: 300, quantum efficiency: 0.9, readout noise: 74.4, spurious charge: 2 × 10^−4^). We simulated 225 000 emitters distributed distrusted over 5000 frames with a minimum distance of 7.5 pixels from each other and an image pixel size of 100 nm. Using our lookup table algorithm, we localized 192 287 (85.5%) of the simulated emitters within nine seconds using our lookup table algorithm, whereas the continuous least-squares fitting algorithm (3D STORM Tools) took 204 seconds to detect 206 976 emitters (92.0%). Localizations around the focus (z-position = 0 nm) had sharper-edged spokes compared to spokes further away from the focus. The reason for this is that photons originating from fluorophores are distributed over a larger pixel area, which results in a lower SNR. Therefore, defocused signals were more challenging to fit because subpixel-determination accuracy is linked directly to the SNR of a signal [28]. However, our lookup table algorithm could handle most of these low SNR signals.

### 3.2. Real-time SMLM

Here, we show the performance of our lookup table algorithm on fixed biological samples. We imaged the actin cytoskeleton of human platelets and human endothelial cells. Real-time localization allowed us to control the 405 nm UV activation laser pulse intensity to regulate the number of active emitters per frame.

**Fig. 2.**
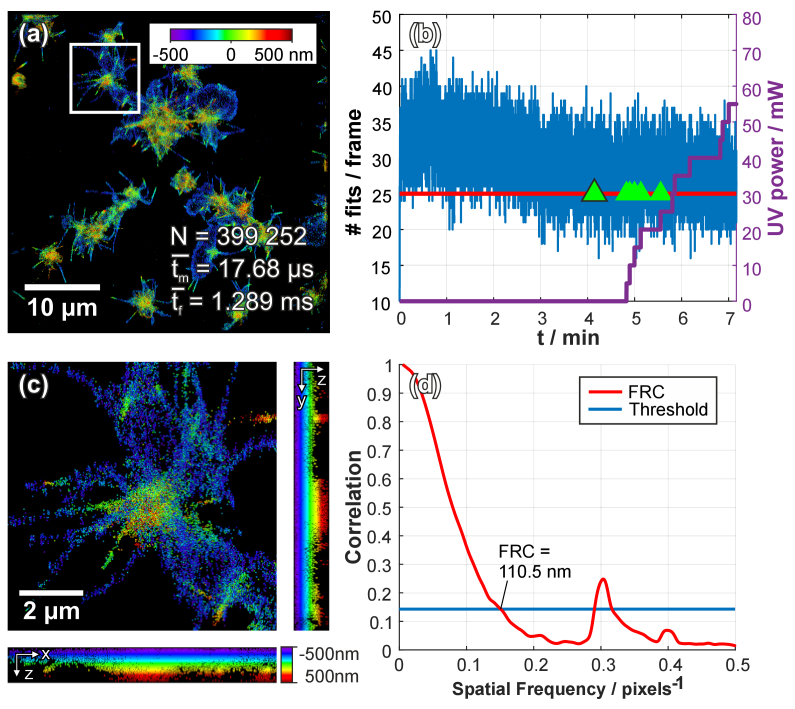
**3D single molecule localization microscopy data of actin cytoskeleton distribution in human platelets analyzed using our real time lookup table algorithm**. The lookup table was populated with templates of 2D elliptical Gaussian functions to approximate the PSF (window size of 9 × 9 pixels). Various lateral positions within the generated template are placed at 0.059 pixel steps (equivalent to 10 nm) in both directions within a range of 4 × 4 pixels around the center. Different shapes of the 2D Gaussian function are generated over a range of 1 000 nm in axial steps of 25 nm. For each combination of the 3D positions, unique template images are calculated. In total 289 296 templates were generated. (a) shows an image of the actin cytoskeleton of fixed human platelets labelled with Phalloidin conjugated Alexa 647 visualized using 3D dSTORM. The emitters of the individual fluorophores were fitted in real-time using the lookup table, where the average time to fit a single emitter signal was *t_m_* = 17.68 µs, a frame including fitting needed on average *t_f_* = 1.289 ms and a total of N = 399 255 emitters was fitted. The experiment consists of 10 000 frames with an ROI of 256 × 256 pixels. (b) The density of active fluorophores is controlled using a 405 nm activation laser pulse. As soon as the number of detected and fitted emitters falls below 25 pre frame, the UV laser power is increased. At the first occurrence, the laser is turned on (indicated by the green triangle with the black border), next, the laser power is increased by 5 mW. (c) shows a magnified area from (**a**) with side projections in XZ and YZ. (**d**) shows a graph of the smoothed Fourier Ring Correlation (FRC) values calculated from two rendered images including localizations of even and odd frames respectively. A FRC of 110.5 nm was calculated by selecting a threshold of “1 over 7” (14.29% correlation). A second crossing of the FRC-threshold can be observed for 57 nm (at 0.292 pixels^-1^).

Platelets, upon activation, reorganize their actin cytoskeleton and thereby change their overall shape. SMLM allows for imagining of the actin cytoskeleton with a resolution of 15–30 nm laterally [29,30 6], which additionally can be extended to the third dimension by using a cylindrical lens introducing astigmatism. We observed the distribution of actin labeled with Alexa Fluor 647, on partial activated and fixed human platelets seeded on a glass surface using 3D dSTORM (see Fig. 2(a)). Thereby, we recorded 10 000 frames with an ROI of 256 × 256 pixels at 25 frames per second and an illumination time of 20 ms. The lookup table for the real-time analysis was populated with templates of 9 × 9 pixels containing 2D elliptical Gaussian functions approximating the PSF. Lateral positions of the elliptical Gaussian functions were varied over a range of four pixels in both directions around the center (in 0.059 pixel steps which equals 10 nm steps) and in the axial direction we generated different shapes corresponding to axial positions over a range of 1000 nm in 25 nm z-steps. In total 106 641 different templates were generated consuming 264 MB of RAM (including the derivations). Our lookup table algorithm detected 399 252 emitters during the acquisition of the experiment and performed the analysis on average in 1.3 ± 0.2 ms per frame. Since one frame is acquired in 40 ms, we had sufficient time to additionally render the SMLM image and apply an automatic feedback control for the activation laser pulse in order to increase the number of active fluorophores. Figure 2(b) illustrates the timeline of the dSTORM experiment, showing the number of localized emitters (blue curve) and the activation laser power (violet curve). If the number of localized emitters falls below a threshold (here we used 25 localizations) over five consecutive frames, we increased the laser power by 5 mW. Only for the first occurrence of this trigger (indicated with a green triangle with back border in Fig. 2(b)), we turned on the UV laser.

We compared the result of our lookup table algorithm with a continuous least-squares fitting algorithm (3D STORM tools). The continuous algorithm needed 269 seconds to analyze 10 000 frames and detected 574 899 localizations by utilizing four (+4 virtual) CPU cores, whereas our lookup table algorithm required only 45 seconds (loading times of the sequences are not included and are dependent on the file format and hard drive speed). Furthermore, we compared the achieved image resolution by calculation of the FRC on the reconstructed localization microscopy image. The FRC (or spectral SNR) is a measurement for image-resolution of diffraction-unlimited images that take both localization precision and the density of rendered localizations into account [30]. In order to calculate the FRC, we rendered two images using 2D symmetrical Gaussian functions with a sigma of 25 nm and a pixel size of 16.67 nm. For each algorithm, we split the localization microscopy dataset into even and odd frames and analyzed the FRC from these two images using the ImageJ plugin FIRE (Fourier Image REsolution) from [30]. The FRC for the lookup table algorithm was 110 nm and 59 nm for the continuous least-squares fitting algorithm (using the thresholding method of “1 over 7” and smoothed FRC curves).

In a second experiment, we analyzed the actin cytoskeleton of fixed human endothelial cells via labeling with Alexa Fluor 647. The actin cytoskeleton in ECs is much denser and scattered with small F-actin fibers and therefore challenging to observe with 3D dSTORM [31]. We recorded a 3D dSTORM experiment of endothelial cells’ actin consisting of 10 000 frames with an ROI of 256 × 256 pixels, acquiring 25 frames per second with an illumination time of 20 ms. Using our real-time lookup table algorithm to analyze the SMLM experiment data, we detected 1 232 530 signals (see Fig. 2(c)) with an average localization time of 2.6 ± 1.1 ms per frame. Since the overall density of emitters was very high (mean localization events per frame was 120), the UV laser pulse was only turned on during minute 4 of the acquisition (indicated with a green triangle with back border in Fig. 2(d)) and there was no need to further increase the laser power.

During post-processing, we compared our lookup table algorithm with a continuous least-squares fitting algorithm (3D STORM Tools [17]) for spatial and temporal performance. Our lookup table algorithm required only 58 seconds to analyze 10 000 frames on a single CPU core, whereas the continuous least-squares algorithm needed 269 seconds on four (+4 virtual) CPU cores and detected 1 287 725 emitters (loading times of the sequences are not included and are dependent on the file format and hard drive speed). Again, we compared the spatial image resolution using FRC for both algorithms. The FRC for the lookup table algorithm was 40 nm, whereas the continuous least-squares SMLM fitting algorithm FRC was 59 nm (using the thresholding method of “1 over 7” and smoothed FRC curves).

Real-time SMLM image rendering was achieved by an improved histogram rendering approach. Newly fitted emitters were binned in a 2D image histogram (e.g. an image is by default 10 times the size of the input frame) and the corresponding pixel was assigned the value of the axial position (the histogram image is initialized with all zeros). If the pixel value of that position was not zero, the highest axial position was kept. In a second step, the histogram image is rendered every 10 frames, because rendering is computationally expensive (rendering time: 23.3 ± 5.7 ms for 2560 × 2560 pixels). The axial positions were color-coded (e.g. rainbow color table) and a Gaussian gradient was drawn around the every non-zero pixels. If the current pixel to render contained no localization (i.e. is zero), the surrounding eight pixels were checked for localizations. If any surrounding pixel contained a localization – depending on the position of current pixel (corner or next to the surrounding pixel) – the color mapped to the axial positon of the localization of the surrounding nonzero pixel is drawn with a Gaussian gradient (e.g. sigma of 0.5 or 1 pixel).

## 4. Discussion

We present a 3D real-time SMLM fitting algorithm that accelerates emitter localization (>10 times) compared to our previously published fitting algorithms [17]. The previously published method uses an unconstrained least-square minimization algorithm for 3D single emitter localization (Double Dogleg optimization [32]) to directly fit a 2D elliptical Gaussian model. However, the algorithm proposed in this paper can only fit the emitter 3D position at discrete steps based on the parameter used for lookup table generation.

Our algorithm is independent from GPU acceleration or multithreading and runs on a single CPU thread. This is possible by using lookup tables containing template images that approximate the PSF of single molecule emitters. The discrete axial and lateral positions at which the different templates are generated stabilizes the fitting procedure. This discretization allows us to only calculate five iterations to find the best fit and avoid local minima. Additionally, we show that decreasing the step size of 3D positions for template generation does not have a major impact on fitting accuracy. Our default step sizes of 10 nm laterally and 25 nm axially are below the Nyquist theorem limit. Typical position accuracies of ∼30 nm laterally and ∼60 nm axially of a 3D SMLM experiment are more than twice the size of our default step sizes. The discrete spacing can only be observed if the render pixel size is less than the lateral step size used for the positions of the generated template images.

**Fig. 3.**
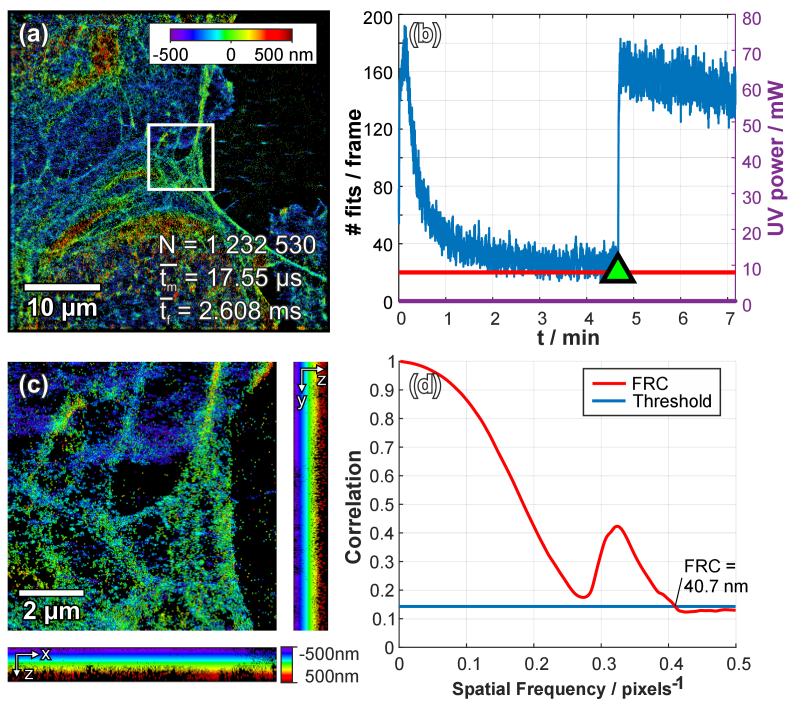
**3D single molecule localization microscopy data of the actin cytoskeleton labeled in human endothelial cells analyzed using our real time lookup table algorithm**. The lookup table was populated with templates of 2D elliptical Gaussian functions which approximate the PSF, with a window size of 9 pixels. Different lateral positions within the generated template are placed at 0.059 pixel steps (equivalent to 10 nm) in both directions within a range of 4 × 4 pixels around the center. Different shapes of the 2D Gaussian function are generated over a range of 1 000 nm in axial steps of 25 nm. For each combination of the 3D positions, unique template images are calculated. In total 289 296 templates were generated. (a) shows an image of the actin cytoskeleton of primary human endothelial cells (phEC) on a gelatine coated glass slide labelled with Phalloidin conjugated Alexa 647. The lookup table algorithm was used to determine emitter positions in real-time, where an average time to fit a single emitter signal was *t_m_* = 1.55 µs, a frame including fitting needed on average *t_f_* = 2.609 ms and a total of N = 1 232 530 emitters was fitted. The experiment consisted of 10 000 frames with an ROI of 256 × 256 pixels. (b) The number of detected emitters per frame was used to control the UV activation laser pulse. Here, the density of fitted localizations was overall high (compared to the platelet experiment), thus the laser pulse was only turned on (indicated by the green triangle with the black border) to keep the number of active fluorophores above 25 per frame. The increase of the active fluorophores after laser activation is caused by UV laser emission even at 0-power adjustment. (c) shows a magnified box area in (**a**) with side projections in XZ and YZ. (**d**) shows a graph of the smoothed Fourier Ring Correlation (FRC) values calculated from two rendered images consisting of localizations of even and odd frames respectively. A FRC of 40.7 nm was calculated by selecting a threshold of “1 over 7” (14.29% correlation). A prior local minimum is visible at 61 nm (at 0.273 pixels^-1^) in (**d**), which is slightly above the threshold. This might indicate the real image resolution and would be consistent with the FRC behavior observed in the previous sample (see Fig. 2(d)).

In Fig. 3(d) we calculated a spatial image resolution of 40.7 nm using FRC, however the FRC curve shows a prior local minimum at 61 nm (at 0.273 pixels^-1^) which is slightly above the threshold. Moreover, in Fig. 2(d) the FRC curve shows a local minimum, which drops below the threshold. Since both curves follow this course, we assume that the found spatial image resolution in Fig. 3(d) is rather 61 nm instead of the 40.7 nm, which is closer to the real FRC value. The additional peaks in Fig. 2(d) and Fig. 3(d) could be a result of pixilation due to our discrete emitter fitting algorithm.

Next, UV-light modulation enables to control the photochemical properties of the fluorophores [6,10], inducing changes in the on/off state duration. A feedback loop, which allows real-time adaptation of the UV-laser power dependent on the number of emitters detected in an imaged frame helps to keep the numbers of emitters constant. Adjustment of emitter numbers is crucial in terms of multi-emitter fitting, optimization of acquisition time or fluorophore blinking properties, etc.

We want to discuss some improvement on how the lookup table algorithm could be extended in the future: 3D emitter PSFs modulated by a cylindrical lens are not the only PSFs localizable by our methods. The flexibility of our lookup table template image generation allows for arbitrary PSFs. For example, PSFs modeled by a phase mask could be used to fill the lookup table. Fitting of phase mask modeled PSFs is computationally demanding (Fourier transformation) even with GPU parallelization [33]. Therefore, our lookup table algorithm could be used to fit emitters in real-time with an improved model if the phase mask is known. Parameters needed for the phase mask model could be calculated from a phase retrieval calibration using Zernike polynomials from a z-stack of fluorescent beads. These parameters can then be used to populate our lookup table with phase mask modeled PSFs at discrete 3D steps.

The temporal performance of our algorithm for real-time emitter localization allows us to use the dwell time until the next image is acquired for additional tasks. Automated control of the number of on-state fluorophores using a UV activation laser pulse and a real-time rendering system, all running in the dwell time of image acquisition, is doable. Furthermore, FRC can be used to determine the SMLM image-resolution and stop image acquisition if the detail density does not continue to increase. FRC allows for the calculation of image-resolution which also takes emitter density into account. FRC requires two rendered SMLM images for image-resolution calculations [31]. This can be implemented by rendering two additional SMLM images, where newly localized emitters are distributed between these two SMLM images and subsequent calculation of the FRC; our algorithm is capable of performing this action in real-time.

Additionally, image quality can be improved by actively controlling the excitation laser power and imaging frame rate based on the real-time localization information of newly analyzed frames. Fluorophore blinking kinetics as well as the initial switch-off phase (in which fluorophores transition to their dark-state) are crucial for (d)STORM experiment’s SMLM image quality. Our real-time algorithm could be used to determine the duration of the initial switch-off phase. As previously stated [6], the excitation laser intensity should be as low as possible to prevent fluorophore bleaching. Only if fluorophores blink uniformly and single emitters be distinguished from each other, then the excitation laser power can be increased to enhance fluorophore blinking. The excitation laser power and the imaging frame rate can be adapted to optimize the photon emission of fluorophores so that their reappearance in subsequent images is minimized – emitter reappearance can be examined by real-time localization information over consecutive frames.

A major challenge in SMLM is the differentiation between true emitters and falsely identified ones. One possibility to distinguish between true emitters and background noise is to use an intensity threshold. However, selecting an intensity threshold is subjective and can vary between experiments and fluorophores. Our algorithm allows for threshold adaptation via user input during the experiment. Thus, threshold selection is subjective. An automated threshold algorithm would be an improvement – e.g. Bayesian thresholding [34]. For Bayesian thresholding a histogram of localized emitter intensities from a defined number of images is calculated. To adapt the threshold, the histogram is analyzed using a Generalized Minimum Error Thresholding algorithm (GMET). This can all be done in real-time since intensities for localized emitters are continuously available during the acquisition.

Another effect influencing SMLM image quality is sample drift introduced by the excitation laser, temperature changes, mechanical relaxation, objective oil expansion/relaxation, or electrical noise. The mechanical displacement accumulates over the long acquisition time of SMLM experiments and impairs the SMLM’s image quality [35]. Currently, two main drift correction/estimation methods are used: The first uses fiducial markers or special hardware to measure the drift directly [36]. The second uses the fitted emitter localizations to directly calculate the drift. The majority of these algorithms use cross-correlation on substacks of SMLM images binned into time intervals of equal length. An alternative to cross-correlation is the direct calculation of the drift from the positions of fitted emitters, avoiding the rendering of multiple SMLM images. As stated [37], drift correction equations can be solved numerically based on distance matrices consisting of fitted emitter positions at different time intervals. However, only a few of these algorithms allow for real-time drift correction. Our algorithm can be used to calculate either the SMLM images used for cross-correlation or directly supply a drift correction algorithm with the currently fitted localizations. Furthermore, these drift estimates can be used to counteract the drift by controlling a 3D piezo positioning stage parallel to the SMLM experiment acquisition.

In conclusion, our algorithm can be used for feedback-controlled real-time SMLM experiments and allows to improve the experiment’s image quality based on real-time localization information. Therefore we supply an example implementation for ImageJ [38] that uses a CPP library for the time-critical algorithms and an easy to extend java interface for expansion and custom lookup table templates.

## Data Availability

Data and source code underlying the results presented in this paper are available at [38].

## Supplemental document

See Supplement 1 for supporting content.
