# Supplementary material for: Real-time 3D single-molecule localization microscopy analysis using lookup tables
Source: Biomed Opt Express. 2021 Jul 16;12(8):4955–68. doi: 10.1364/BOE.424016 (PMC8407837; doi:10.1364/BOE.424016)
Supplement: Supplementary file 2 [file boe-12-8-4955-s001.pdf]

Supplemental Document

Biomedical Optics EXPRESS

Real-time 3D single-molecule localization microscopy analysis using lookup tables: supplement

FABIAN HAUSER[1,2,*] AND JAROSLAW JACAK[1,2]

[1] *University of Applied Sciences, Upper Austria School of Medical Engineering and Applied Social Sciences, Garnisonstraße 21, 4020 Linz, Austria*
[2] *Austrian Cluster for Tissue Regeneration, Vienna 1200, Austria*
[*] *fabian.hauser@fh-linz.at*

This supplement published with The Optical Society on 16 July 2021 by The Authors under the terms of the Creative Commons Attribution 4.0 License in the format provided by the authors and unedited. Further distribution of this work must maintain attribution to the author(s) and the published article's title, journal citation, and DOI.

Supplement DOI: https://doi.org/10.6084/m9.figshare.14813070

Parent Article DOI: https://doi.org/10.1364/BOE.424016

Real-Time 3D Single-Molecule Localization Microscopy Analysis Using Lookup Tables: Supplement

Fabian Hauser [1,2,*] and Jaroslaw Jacak [1,2]

[1]University of Applied Sciences, Upper Austria School of Medical Engineering and Applied Social Sciences, Garnisonstraße 21, 4020 Linz, Austria

[2]Austrian Cluster for Tissue Regeneration, Vienna 1200, Austria

**fabian.hauser@fh-linz.at*

1. Supplementary Images

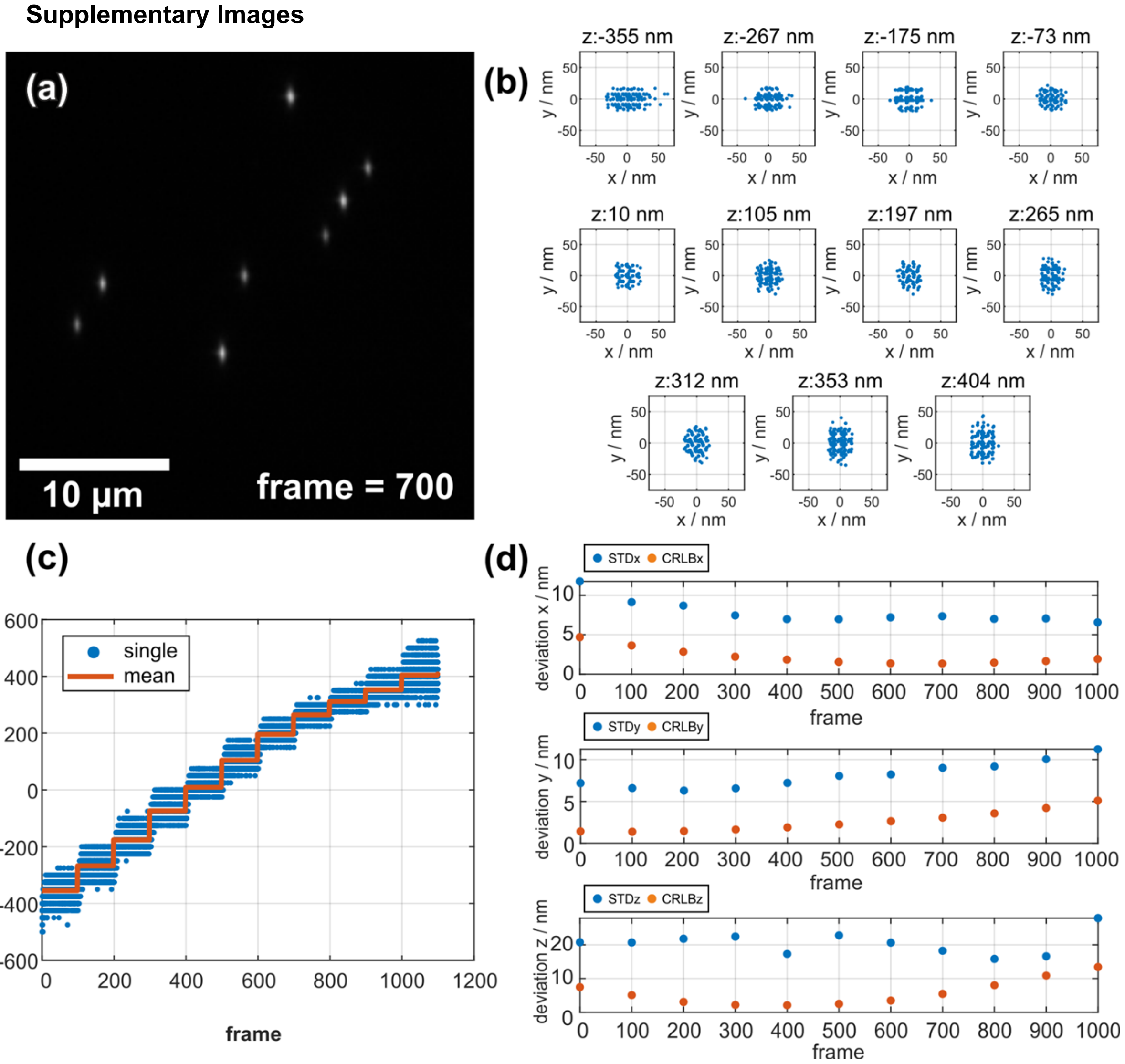

Fig. S1. Determination of positional accuracy and comparison with Cramér–Rao lower bound (CRLB). Images of fluorescent beads (TetraSpeck™ Microspheres, 0.1 µm) were acquired while changing the axial position of the focal plane ($\Delta z = 100$ nm) from -500 nm to -500 nm. (**a**) shows a single frame of by astigmatism distorted signals of individual beads (200 nm above plane of maximum focus). 100 frames were acquired in each plane for calculation of the deviation of (xyz)-position. (**b**) shows deviation in the (xyz)-position and the mean axial positon retrieved from 3D localized beads using our lookup table based algorithm (window size 11 pixels, $\Delta xy = 0.1$ pixels, $r_{xy} = 4$ pixels, $\Delta z = 25$ nm, $r_z = 1250$ nm, threshold: 175). (**c**) shows z-positions found by our algorithm versus the number of acquired frames (frame 0 to 99 → z = -500 nm, frame 1000 to 1099 → z = +500 nm). (**d**) Comparison of the lowest possible positional accuracy (determined via CRLB) to the experimentally obtained deviation in (xyz)-position.

2. Complete models

Transformation of the ith and j[th] pixel and the models xy position by multiplication with a rotation matrix. The values t_x and t_y are the rotated positions used for the model. The angle φ is obtained from a calibration to determine sigmas from different axial positions of astigmatism point spread functions (PSFs).

$$\begin{pmatrix} t_x \\ t_y \end{pmatrix} = \begin{bmatrix} \cos(\varphi) & \sin(\varphi) \\ -\sin(\varphi) & \cos(\varphi) \end{bmatrix} \begin{pmatrix} i-x \\ j-y \end{pmatrix}$$

$$t_x = (i-x)\cos(\varphi) + (j-y)\sin(\varphi) \tag{S1}$$

$$t_y = -(i-x)\sin(\varphi) + (j-y)\cos(\varphi)$$

2.1 2D elliptical Gaussian model

$$PSF_{i,j}(x,y,z,\varphi) = \exp\left(-\frac{t_x^{\,2}}{2\sigma_x(z)^2} - \frac{t_y^{\,2}}{2\sigma_y(z)^2} \right)$$

$$\frac{\partial PSF_{i,j}(x,y,z,\varphi)}{\partial x} = \left(\frac{t_x\cos(\varphi)}{\sigma_x(z)^2} - \frac{t_y\sin(\varphi)}{\sigma_y(z)^2} \right) \exp\left(-\frac{t_x^{\,2}}{2\sigma_x(z)^2} - \frac{t_y^{\,2}}{2\sigma_y(z)^2} \right)$$

$$\frac{\partial PSF_{i,j}(x,y,z,\varphi)}{\partial y} = \left(\frac{t_x\sin(\varphi)}{\sigma_x(z)^2} - \frac{t_y\cos(\varphi)}{\sigma_y(z)^2} \right) \exp\left(-\frac{t_x^{\,2}}{2\sigma_x(z)^2} - \frac{t_y^{\,2}}{2\sigma_y(z)^2} \right) \tag{S2}$$

$$\frac{\partial PSF_{i,j}(x,y,z,\varphi)}{\partial z} = \left(\frac{t_x^{\,2}\cdot\partial\sigma_x(z)/\partial z}{\sigma_x(z)^3} + \frac{t_y^{\,2}\cdot\partial\sigma_y(z)/\partial z}{\sigma_y(z)^3} \right) \exp\left(-\frac{t_x^{\,2}}{2\sigma_x(z)^2} - \frac{t_y^{\,2}}{2\sigma_y(z)^2} \right)$$

2.2 Integrated 2D elliptical Gaussian model

Transformation of the ith and j[th] pixel and the models xy position by multiplication with a rotation matrix. The values t_x and t_y are the rotated positions used for the model (see equation S1). Erf(x) is the so called "Gaussian error function", which is the integral a normal distribution (special sigmoid function).

$$
\begin{aligned}
dE_x &= 0.5\cdot \operatorname{erf}\left(\frac{t_x+0.5}{\sqrt{2}\sigma_x(z)}\right) - 0.5\cdot \operatorname{erf}\left(\frac{t_x-0.5}{\sqrt{2}\sigma_x(z)}\right)\\
dE_y &= 0.5\cdot \operatorname{erf}\left(\frac{t_y+0.5}{\sqrt{2}\sigma_y(z)}\right) - 0.5\cdot \operatorname{erf}\left(\frac{t_y-0.5}{\sqrt{2}\sigma_y(z)}\right)
\end{aligned} \tag{S3}
$$

$$
\begin{aligned}
PSF_{i,j}(x,y,z,\varphi) &= 2\pi\sigma_x(z)\sigma_y(z)dE_x dE_y\\
\frac{\partial PSF_{i,j}(x,y,z,\varphi)}{\partial x} &= \sqrt{2\pi}\sigma_y\cos(\varphi)dE_y\left[\exp\left(-\frac{(t_x-0.5)^2}{2\sigma_x^{\,2}}\right)-\exp\left(-\frac{(t_x+0.5)^2}{2\sigma_x(z)^2}\right)\right]\\
&\quad -\sqrt{2\pi}\sigma_x\sin(\varphi)dE_x\left[\exp\left(-\frac{(t_y-0.5)^2}{2\sigma_y^{\,2}}\right)-\exp\left(-\frac{(t_y+0.5)^2}{2\sigma_y^{\,2}}\right)\right]\\
\frac{\partial PSF_{i,j}(x,y,z,\varphi)}{\partial y} &= \sqrt{2\pi}\sigma_y\sin(\varphi)dE_y\left[\exp\left(-\frac{(t_x-0.5)^2}{2\sigma_x^{\,2}}\right)-\exp\left(-\frac{(t_x+0.5)^2}{2\sigma_x^{\,2}}\right)\right]\\
&\quad +\sqrt{2\pi}\sigma_x\cos(\varphi)dE_x\left[\exp\left(-\frac{(t_y-0.5)^2}{2\sigma_y^{\,2}}\right)-\exp\left(-\frac{(t_y+0.5)^2}{2\sigma_y^{\,2}}\right)\right]\\
\frac{\partial PSF_{i,j}(x,y,z,\varphi)}{\partial z} &= \frac{\sqrt{2\pi}\sigma_y dE_y}{\sigma_x}\frac{\partial\sigma_x}{\partial z}\left[\begin{array}{l}(t_x-0.5)\exp\left(-\frac{(t_x-0.5)^2}{2\sigma_x^{\,2}}\right)-\\ (t_x+0.5)\exp\left(-\frac{(t_x+0.5)^2}{2\sigma_x^{\,2}}\right)\end{array}\right]\\
&\quad +\frac{\sqrt{2\pi}\sigma_x dE_x}{\sigma_y}\frac{\partial\sigma_y}{\partial z}\left[\begin{array}{l}(t_y-0.5)\exp\left(-\frac{(t_y-0.5)^2}{2\sigma_y^{\,2}}\right)-\\ (t_y+0.5)\exp\left(-\frac{(t_y+0.5)^2}{2\sigma_y^{\,2}}\right)\end{array}\right]\\
&\quad +2\pi dE_x dE_y\left(\sigma_x\frac{\partial\sigma_y}{\partial z}+\frac{\partial\sigma_x}{\partial z}\sigma_y\right)
\end{aligned} \tag{S4}
$$
